# Knowledge, attitude, and practice toward postoperative self‐management among patients after percutaneous coronary intervention: A structural equation modeling analysis

**DOI:** 10.1002/clc.24232

**Published:** 2024-03-15

**Authors:** Hailing Lei, Lin Zhu, Xin Zhang

**Affiliations:** ^1^ Department of Cardiology, Beijing Jishuitan Hospital Capital Medical University Beijing China

**Keywords:** knowledge, attitude, practice, percutaneous coronary intervention, postoperative, self‐management

## Abstract

**Objective:**

The knowledge, attitude, and practice (KAP) toward post‐percutaneous coronary intervention (PCI) self‐management among Chinese patients remains unknown. This study investigated the KAP toward postoperative self‐management among patients after PCI.

**Hypothesis:**

Patients exhibit poor knowledge, attitudes, and practices regarding post‐PCI self‐management, requiring enhanced education strategies.

**Methods:**

This cross‐sectional study recruited patients after PCI at Jishuitan Hospital, Beijing, between November 2022 and May 2023. Inclusion criteria comprised patients 1–3 months post‐PCI, those capable of self‐care, and those willingly participating. The questionnaire (49 items) was designed with reference to current guidelines (the Cronbach *α* = .829). The final questionnaire included four dimensions with 49 items. The Pearson correlation analysis and structural equation modeling (SEM) were used to determine the relationship among knowledge, attitude, and practice.

**Results:**

A total of 476 valid questionnaires were included. The knowledge, attitude, and practice scores were 8.24 ± 2.78 (possible range: 0–12), 21.61 ± 3.15 (possible range: 9–45), and 32.62 ± 3.75 (possible range: 10–50). The Pearson correlation analysis showed only knowledge scores were correlated with the attitude scores (*r* = .446, *p* < .001). The SEM showed that knowledge directly affects attitude (*β* = .616, *p* < .001) but had no influence on practice (*β* = .119, *p* = .155); attitude had no influence on practice (*β* = .015, *p* = .809).

**Conclusion:**

This study indicated that patients had poor knowledge, unfavorable attitudes, and unsatisfied practice toward post‐PCI self‐management. Strengthening patient health education through diverse approaches is imperative.

## Graphical abstract

Patients after percutaneous coronary intervention (PCI) exhibit deficient knowledge, negative attitudes, and inadequate practices in self‐management. While knowledge significantly influences attitude, its impact on practice is limited. Strengthening diverse health education strategies is crucial for enhancing post‐PCI self‐management.

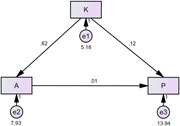

## 1 BACKGROUND

Self‐management involves active self‐behavior management and supervision to prevent disease progression and includes medication adherence, physical activity, and diet.^1^ A meta‐analysis revealed that patients who underwent percutaneous coronary intervention (PCI) and had good self‐management of physical activity had a 13% lower overall mortality and 26% lower cardiac mortality 24 months after PCI.^2^ Still, self‐management can vary among patients due to different situations, conditions, comorbidities, socioeconomic statuses, and so forth, and a specific patient with coronary artery disease (CAD) can also have evolution or devolution in self‐management over the acute and long‐term chronic stages.^3^

After PCI, self‐management requires specific knowledge about a healthy lifestyle, physical activity, diet, warning signs, and laboratory results.^4^, ^5^ Knowledge, attitude, and practice (KAP) surveys are cross‐sectional studies that provide qualitative and quantitative data about the possible gaps and barriers to implementing a specific subject in a specific population.^6^, ^7^ KAP studies are particularly useful for designing future education interventions in the surveyed populations. A study in Ethiopia showed that patients with acute coronary syndromes (ACS) (which includes ST‐elevation myocardial infarction [STEMI]) had improper knowledge, unfavorable attitudes, and improper beliefs regarding their disease and its management.^8^ A study revealed a high degree of adherence to post‐PCI discharge recommendations but a limited understanding of the disease among patients who underwent PCI.^9^ Another study revealed that the knowledge and awareness levels of patients who underwent PCI were unrelated to the risk factor profiles and that attendance was low at cardiac rehabilitation clinics.^10^ The data on Chinese patients are limited. Therefore, this study aimed to investigate the KAP toward postoperative self‐management among patients after PCI.

## 2 METHODS

### 2.1 Study design and participants

This cross‐sectional study recruited patients after PCI at Jishuitan Hospital, Beijing, between November 2022 and May 2023. Jishuitan Hospital covers three districts in Beijing and admits over 1300 patients per year for PCI. The inclusion criteria were (1) patients who were 1 month to 3 months after PCI, (2) patients who could take care of themselves, and (3) patients who volunteered to participate in the study. The exclusion criteria were (1) consciousness barriers, or (2) lack of the ability to learn or self‐manage. The study was approved by the ethics committee of Jishuitan Hospital, Beijing (Approval No. 2022‐185). All participants provided written informed consent before completing the survey.

### 2.2 Questionnaire

The questionnaire was designed with reference to the Chinese Guidelines for Percutaneous Coronary Intervention,^11^ the Chinese Expert Consensus on Blood Pressure Management after Percutaneous Coronary Intervention,^12^ and the Consensus on Perioperative Myocardial Injury and Infarction Associated with PCI and Prognosis (ESC/EAPCI Consensus).^13^ The questionnaire was reviewed by five cardiologists with more than 20 years of experience. A pretest was conducted (*n* = 30), and the Cronbach *α* was 0.829, indicating good internal consistency. Reliability testing was conducted through Cronbach calculations *α* reliability coefficient. The formula for calculating the Cronbach *α* coefficient is

$$\alpha =\left(k/\left(k-1\right)\right)\times \left(1-\left(\Sigma S^{2\_I}/s^{2\_t}\right)\right)$$

where *k* is the number of items in the scale, s^2_I^ is the variance of the *i*th item, and s^2_*t*^ is the total variance of the scale.

This formula involves the ratio of the number of items to the number of items minus 1, multiplied by the difference between the total variance of the scale and the variance of each item. The Cronbach *α* coefficient ranges from 0 to 1, with higher values indicating higher internal consistency.

The final questionnaire included four dimensions with 49 items. The basic characteristics consisted of 15 items; the knowledge dimension consisted of 12 items. The attitude dimension consisted of 10 items. The practice dimension consisted of 12 items. The knowledge items were scored 1 for correct answers and 0 for wrong or unclear answers, with a possible score ranging from 0 to 12 points. The attitude and practice items were mainly scored on a five‐point Likert scale, ranging from very positive (5) to very negative (1), with item A8 having no obvious positive or negative tendencies, so no score was assigned, and only descriptive statistics were used for this item. Item P4 and P12 were semi‐open questions and were also only given descriptive statistics. The possible score range for the attitude dimension was 9–45 points, and for the practice dimension was 10–50 points. For all dimensions, scores ≥70% were considered adequate knowledge, positive attitude, and proactive practice.^14^

The questionnaires were advertised, and the participants were recruited by convenience sampling. The participants were the inpatients in the cardiology ward of our hospital. After identifying the eligible patients and excluding those meeting the exclusion criteria, the nurses pushed the questionnaire to the patients after explaining the study and answering their questions. The patient scanned the QR code to access the questionnaire. The first step was to sign the informed consent form, which then unlocked the questionnaire itself. The patients were approached for participation 1–3 months after PCI during a follow‐up visit. The data were collected using an electronic questionnaire on the Sojump online platform in China. To avoid repetition, IP restriction was applied, which meant that the survey could only be completed once from a single IP address. The duty nurse was responsible for collecting the questionnaires. If a participant had problems, the nurse was also responsible for answering the questions in a timely manner. After data collection was completed, the questionnaires were checked for quality by members of the research team. An incomplete questionnaire, obvious logical errors, or a pattern of answering by selecting the same option for all items was considered invalid.

### 2.3 Statistical analysis

SPSS 22 (IBM Corp.) was used for statistical analysis. The continuous variables were expressed using mean ± standard deviation (SD) and analyzed using the *t* test or one‐way ANOVA. The Bonferroni method was used for post hoc comparisons. The categorical variables were expressed as *n* (%) and analyzed using the *χ* ^2^ test. The Pearson correlation analysis was used to analyze the correlation between knowledge, attitude, and practice. The interactions among the KAP dimensions were evaluated using a structural equation model (SEM). It was hypothesized that knowledge directly affects attitude and practice, while attitude directly affects practice. Two‐sided *p* values <.05 were considered statistically significant.

## 3 RESULTS

### 3.1 Characteristics of the participants

**Figure 1:**
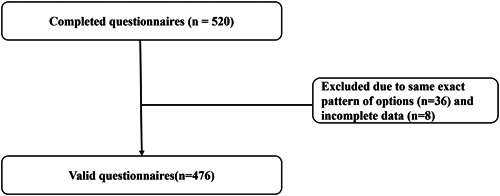
Flow chart of patients.

A total of 520 questionnaires were included; 36 were excluded because of the same exact pattern of options, and eight because of incomplete data. Therefore, 476 valid questionnaires (91.54%) were analyzed (Figure 1). There were two patients with cardiac arrest, one with cerebral embolism, four with gastrointestinal bleeding, 16 with subcutaneous bleeding at the puncture site, and one with gingival bleeding. There were no cases of coronary perforation during the perioperative period. One patient had recurrent myocardial infarction, and one patient had stent thrombosis within 1 month. All patients were treated with a dual anticoagulant regimen of aspirin 100 mg QD clopidogrel 90 mg BID.

Most participants were female (57.56%), ≥70 years (40.3%), nonurban residents (60.92%), junior college/bachelor's degree or above (45.59%), professional and technical staff (42.44%), with an income of 5000–10,000 CNY (700–1400 USD) per month (48.53%), never smoked (45.38%), never drinking (50.21%), and diagnosed with CAD for <6 months (43.28%). Among them, 81.04% were treated with drugs, 11.34% underwent intravenous thrombolysis, and 89.50% underwent PCI. In addition, 67.02% had hypertension, 41.18% had diabetes mellitus, 30.46 had kidney diseases, and 18.90% had thyroid diseases (Table 1).

**Table 1:** Characteristics of the participants.

Variables	*n* (%)	Knowledge score		Attitude score		Practice score	
		Mean ± SD	*p*	Mean ± SD	*p*	Mean ± SD	*p*
Total	476	8.24 ± 2.78		21.61 ± 3.15		32.62 ± 3.75	
Gender			<.001		0.147		.047
Male	202 (42.44)	7.48 ± 2.38		21.36 ± 3.18		32.22 ± 3.68	
Female	274 (57.56)	8.80 ± 2.03		21.78 ± 3.18		32.91 ± 3.78	
Age, years			.711		0.185		.660
40–59	133 (27.9)	8.13 ± 2.57		21.41 ± 3.28		32.43 ± 3.50	
60–69	151 (31.7)	8.35 ± 2.09		21.37 ± 3.15		32.83 ± 3.91	
≥70	192 (40.3)	8.23 ± 2.21		21.93 ± 3.04		32.58 ± 3.80	
Residence			<.001		0.033		.265
Urban	186 (39.08)	9.12 ± 1.43		21.99 ± 3.01		32.85 ± 3.51	
Nonurban (rural/suburban)	290 (60.92)	7.67 ± 2.53		21.36 ± 3.22		32.46 ± 3.89	
Education			<.001		<.001		.512
Middle school and below	167 (35.08)	7.44 ± 1.89		21.15 ± 2.83		32.81 ± 3.72	
High school/technical secondary school	92 (19.33)	7.15 ± 3.22		20.97 ± 3.79		32.25 ± 4.00	
Junior college/bachelor's degree and above	217 (45.59)	9.32 ± 1.45		22.23 ± 2.98		32.62 ± 3.67	
Occupation			<.001		0.027		.608
Professional and technical staff (teachers, doctors, engineers, writers, and other professionals)	202 (42.44)	8.95 ± 2.46		21.87 ± 3.31		32.76 ± 3.81	
Commercial and service industry personnel	98 (20.59)	6.86 ± 1.96		20.86 ± 2.74		32.72 ± 3.75	
Others	176 (36.97)	8.20 ± 1.84		21.72 ± 3.13		32.39 ± 3.69	
Household per capita income (CNY/month)			<.001				.877
2000–5000	113 (23.74)	6.63 ± 1.38		20.23 ± 2.69	<.001	32.55 ± 3.79	
5000–10,000	231 (48.53)	8.60 ± 2.74		21.84 ± 3.26		32.71 ± 3.93	
>10,000	132 (27.73)	8.99 ± 0.98		22.36 ± 2.97		32.52 ± 3.39	
Marital status			.122		0.661		.146
Married	371 (77.94)	8.15 ± 2.36		21.64 ± 3.24		32.48 ± 3.72	
Unmarried/divorced/widowed	105 (22.06)	8.54 ± 1.96		21.49 ± 2.80		33.09 ± 3.82	
Medical insurance			<.001		0.766		.056
Insured	456 (95.80)	8.16 ± 2.30		21.61 ± 3.19		32.68 ± 3.78	
Uninsured	20 (4.20)	10.00 ± 0.00		21.40 ± 2.14		31.05 ± 2.50	
Smoking			<.001		<.001		.792
Never smoked	216 (45.38)	9.46 ± 1.06		21.87 ± 3.11		32.68 ± 3.91	
Used to smoke	91 (19.12)	7.47 ± 3.36		19.59 ± 2.88		32.37 ± 3.98	
Still smoking	169 (35.50)	7.09 ± 1.91		22.34 ± 2.89		32.67 ± 3.42	
Number of drinking times per month			<.001		<.001		.924
0	239 (50.21)	8.93 ± 2.10		22.08 ± 3.34		32.55 ± 3.95	
1–10	156 (32.77)	7.16 ± 2.60		20.79 ± 2.81		32.69 ± 3.53	
≥11	81 (17.02)	8.27 ± 0.94		21.77 ± 2.90		32.67 ± 3.58	
Duration of disease			<.001		<.001		.629
≤6 months	206 (43.28)	8.30 ± 1.77		22.54 ± 3.05		32.79 ± 3.51	
>6 months, ≤1 year	70 (14.71)	9.29 ± 1.08		20.53 ± 2.44		32.77 ± 3.89	
>1 year, ≤3 years	79 (16.60)	10.48 ± 0.50		23.68 ± 2.29		32.58 ± 4.03	
>5 years	121 (25.42)	6.07 ± 2.39		19.28 ± 2.47		32.25 ± 3.89	
Treatment for coronary heart disease (multiple choice)							
Medication	381 (80.04)						
Intravenous thrombolysis	54 (11.34)						
Percutaneous coronary intervention (PCI)	426 (89.50)						
Other	87 (18.28)						
Comorbidity							
Hypertension	319 (67.02)						
Diabetes mellitus	196 (41.18)						
Thyroid disease	90 (18.90)						
Renal disease	145 (30.46)						
Other	108 (22.69)						

### 3.2 Knowledge, attitude, and practice

The knowledge score was 8.24 ± 2.78 (68.67%, possible range: 0‐12), indicating poor knowledge. Higher knowledge scores were observed in females (*p* < .001), urban (*p* < .001), junior college/bachelor's degree and above education (*p* < .001), professional and technical staff (*p* < .001), income >5000 (700 USD) (*p* < .001), without insurance (*p* < .001), never smoked (*p* < .001), never drinking (*p* < .001), and duration of disease of 1.1‐3 years (*p* < .001) (Tables 1 and Supporting Information S1: Table S1). There were several knowledge items with a correct rate lower than 70%: K1 (66.39%; “After PCI for myocardial infarction, care must be taken to prevent recurrence of myocardial infarction.”), K2 (61.55%; “After PCI, patients should carry nitroglycerin with them at all times.”), K3 (12.82%; “Stent thrombosis is a serious complication after PCI, and it was mostly found in the early post‐PCI period (0 to 30 days).”), K6 (28.57%; “If you have no uncomfortable conditions after PCI, you don't need to have regular follow‐up consultations.”), K7 (43.70%; “For patients with a history of hypertension and with persistent systolic blood pressure >130 mmHg after PCI, it should be considered medication to lower blood pressure.”), and K12 (68.10%; “Patients need to observe themselves for symptoms such as vomiting blood, black stools, bleeding spots on the skin and bleeding gums, where the use of anticoagulants after PCI.”) (Supporting Information S1: Table S2).

The main source of information was the medical staff (65.55%), followed by the social network (53.99%), community advertisements (37.18%), the Internet (37.18%), TV (30.88%), and magazines (18.70%) (Figure S1).

The attitude score was 21.61 ± 3.15 (48.02%, possible range: 9–45), indicating an unfavorable attitude. Higher attitude scores were observed in urban residents (*p* = .033), higher education (*p* < .001), professional and technical staff (*p* = .027), higher income (*p* < .001), still smoking (*p* < .001), never drinking (*p* < .001), and 1.1–3 years since diagnosis (*p* < .001) (Table 1 and Supporting Information S1: Table S1). Supporting Information S1: Table S3 shows the distribution of the attitude toward the items.

The practice score was 32.62 ± 3.75 (65.24%, possible range: 10–50), indicating poor practice. Females had higher practice scores (*p* = .047) (Table 1). Supporting Information S1: Table 4 presents the distribution of the practices towards the items. Figure S2 presents the reasons for not attending the follow‐up visits on time: 51.68% for economic reasons, 23.74% for a limited time, 14.29% for thinking that follow‐up is unnecessary, and 5.67% for psychological factors.

### 3.3 Correlations

The knowledge scores were correlated with the attitude scores (*r* = .446, *p* < .001), but there were no correlations between the knowledge and practice scores (*p* = .089) and between the attitude and practice scores (*p* = .330) (Table 2).

**Table 2:** Correlations.

	Knowledge	Attitude	Practice
Knowledge	1		
Attitude	0.446 (*p* < .001)	1	
Practice	0.078 (*p* = .089)	0.045 (*p* = .330)	1

### 3.4 Structural equation modeling

**Figure 2:**
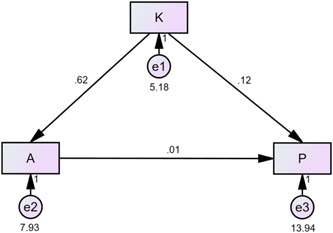
Structural equation modeling.

Figure 2 and Table 3 show that knowledge directly affects attitude (*β* = .616, *p* < .001) but has no influence on practice (*β* = .119, *p* = .155). Attitude had no influence on practice (*β* = .015, *p* = .809).

**Table 3:** Structural equation model (SEM) parameters.

Path			Estimate	*p*
Attitude	←	Knowledge	0.616	<.001
Practice	←	Attitude	0.015	.809
Practice	←	Knowledge	0.119	.155

## 4 DISCUSSION

The results indicated that Chinese patients had poor knowledge, unfavorable attitudes, and poor practice toward post‐PCI self‐management. The SEM showed that knowledge directly affects attitude but has no influence on practice, while attitude had no influence on practice. This study could help design educational interventions for improving the self‐management of the patients after PCI, hence improving their prognosis.

Post‐PCI self‐management is a package of life habits, behaviors, medication adherence, and medical visit adherence that aims to improve the prognosis of the patients.^1^, ^2^ A major limiting factor in the performance of post‐PCI self‐management is the knowledge and correct understanding of the self‐management goal, the actions to take, and events/factors to watch for.^4^, ^5^ It is already well‐known that the quality of self‐management varies widely among individuals due to differences in physical function,^15^ social support,^16^, ^17^ depression/anxiety,^18^ perceived barriers,^17^ and socioeconomic status.^19^, ^20^ Therefore, identifying the factors associated with a lower KAP could help identify the patients in greater need of intervention. Indeed, the present study showed that the participants with a lower socioeconomic status displayed lower KAP scores. Patients living in urban areas also had higher KAP, mainly due to the better healthcare services and community support in urban areas in China.^21^ The participants who never smoked had a better knowledge of post‐PCI self‐management than those who smoked, probably because people who never smoked might have a higher awareness of the possible harms and a higher general knowledge of health. Surprisingly, the patients still smoking had higher attitude scores, possibly because they were aware of cigarette harm to their cardiovascular health and were willing to change their self‐management. The participants with a disease course of 1.1–3 years also displayed higher KAP, possibly because the patients had more time to gain knowledge and change their behaviors after the acute phase of CAD and the immediate period. Still, globally, the present study showed that the patients had a poor KAP towards post‐PCI self‐management. A study from Ethiopia also showed poor KAP in similar patients,^8^ while a study from Canada showed high practice but poor knowledge.^9^ Hence, because of poor knowledge and because knowledge influences attitudes, improving knowledge should help patient self‐management after PCI.

A study showed that the knowledge of ACS symptoms (including STEMI) and risk factors was associated with positive attitudes and beliefs towards treatments and management,^22^ while a lack of knowledge is associated with treatment delays.^23^, ^24^ A study in China revealed poor adherence to self‐management after PCI, and that knowledge was independently associated with adherence.^25^ In the present study, knowledge was only correlated with attitude, and neither knowledge nor attitude was correlated with practice. The SEM showed similar results. Life habits like diet, smoking, and drinking are notoriously hard to change, even in patients who underwent life‐threatening events such as STEMI and PCI.^26^ Nevertheless, studies showed that education could improve self‐management after PCI.^22^, ^27^ Unfortunately, self‐management intervention studies are often biased because they usually include patients already willing to improve their self‐management, while those unwilling to improve their self‐management usually refuse to participate. Nevertheless, the present study identified several areas with poor knowledge, including the possibility of CAD recurrence, carrying nitroglycerin, the risk of in‐stent thrombosis, the need for consulting in the presence of symptoms, controlling blood pressure, and watching for side effects of anticoagulants. Education programs should be designed to improve the patients' knowledge about self‐management. Whether it could translate into better practice should be evaluated.

This study had limitations. It was a single‐center study that enrolled participants from a relative area, resulting in a small sample size with low generalizability. The questionnaire was designed by local investigators according to the local practice, guidelines, and policies, and it might not be exportable or applicable to other centers. KAP surveys are a snapshot of a specific subject in a specific population at a specific point in time. Therefore, it can be used to examine the factors associated with KAP, but not the risk factors for poor KAP or the evolution of KAP in time. Nevertheless, the results can be used as a baseline to examine the effect of future KAP interventions in the same area. Finally, all KAP analyses are subjected to the social desirability bias, which entails that some participants can be tempted to answer what they know to be the right answer or behavior instead of what they really do.^28^, ^29^

In conclusion, the results indicated that Chinese patients had poor knowledge, unfavorable attitudes, and low practice towards post‐PCI self‐management. Knowledge directly affects attitude but has no influence on practice, while attitude has no influence on practice. Interventions should be designed to improve the KAP about post‐PCI self‐management in patients after PCI, which could help improve their prognosis.

## AUTHOR CONTRIBUTIONS

Hailing Lei and Lin Zhu carried out the studies, participated in collecting data, and drafted the manuscript. Hailing Lei and Xin Zhang, Lin Zhu performed the statistical analysis and participated in its design. All authors read and approved the final manuscript.

## CONFLICT OF INTEREST STATEMENT

The authors declare no conflicts of interest.

## Supporting information

Supporting information.

Supplement Figure 1. Ways to acquire knowledge.

Supplement Figure 2. Possible reasons for not being able to return to the hospital on time.

## Data Availability

The datasets used and/or analyzed during the current study are available from the corresponding author upon reasonable request.
