# Supplementary material for: Knowledge, attitude, and practice toward postoperative self‐management among patients after percutaneous coronary intervention: A structural equation modeling analysis
Source: Clin Cardiol. 2024 Mar 15;47(3):e24232. doi: 10.1002/clc.24232 (PMC10943248; doi:10.1002/clc.24232)
Supplement: Supplementary file 1 — Supporting information. [file CLC-47-e24232-s003.docx]

# Supplementary Table 1. Bonferroni post hoc analysis

**Knowledge Dimension**

|  | P |
| --- | --- |
| Education: Middle School and below vs. High school/Technical secondary school | 0.858 |
| Education: Middle School and below vs. Junior college/Bachelor’s degree and above | < 0.05 |
| Education: Junior college/Bachelor’s degree and above vs. High school/Technical secondary school | < 0.05 |
| Occupation: Professional and technical staff vs. Commercial and service industry personnel | < 0.05 |
| Occupation: Professional and technical staff vs. Others | 0.002 |
| Occupation: Commercial and service industry personnel vs. Others | < 0.05 |
| Household per capita income: 2000-5000 vs. 5000-10,000 | < 0.05 |
| Household per capita income: 2000-5000 vs. >10,000 | < 0.05 |
| Household per capita income: 5000-10,000 vs. >10,000 | 0.252 |
| Smoking status: Never smoked vs. Used to smoke | < 0.05 |
| Smoking status: Never smoked vs. Still smoking | < 0.05 |
| Smoking status: Used to smoke vs. Still smoking | 0.433 |
| Times of alcohol consuming: 0 vs. 1-10 | < 0.05 |
| Times of alcohol consuming: 0 vs. ≥11 | 0.050 |
| Times of alcohol consuming: 1-10 vs. ≥11 | 0.001 |
| How long has it been since you were first diagnosed with coronary heart disease: ≤6 months vs. >6 months, ≤1 year | < 0.05 |
| How long has it been since you were first diagnosed with coronary heart disease: ≤6 months vs. >1 year, ≤3 years | < 0.05 |
| How long has it been since you were first diagnosed with coronary heart disease: ≤6 months vs. >5 years | < 0.05 |
| How long has it been since you were first diagnosed with coronary heart disease: >6 months, ≤1 year vs. >1 year, ≤3 years | < 0.05 |
| How long has it been since you were first diagnosed with coronary heart disease: >6 months, ≤1 year vs. >5 years | < 0.05 |
| How long has it been since you were first diagnosed with coronary heart disease: >1 year, ≤3 years vs. >5 years | < 0.05 |

**Attitude dimension**

|  | P |
| --- | --- |
| Education: Middle School and below vs. High school/Technical secondary school | > 0.999 |
| Education: Middle School and below vs. Junior college/Bachelor’s degree and above | 0.002 |
| Education: Junior college/Bachelor’s degree and above vs. High school/Technical secondary school | 0.004 |
| Household per capita income: 2000-5000 vs. 5000-10000 | < 0.05 |
| Household per capita income: 2000-5000 vs. >10000 | < 0.05 |
| Household per capita income: 5000-10000 vs. >10000 | 0.358 |
| Smoking status: Never smoked vs. Used to smoke | < 0.05 |
| Smoking status: Never smoked vs. Still smoking | 0.385 |
| Smoking status: Used to smoke vs. Still smoking | < 0.05 |
| Times of alcohol consuming: 0 vs. 1-10 | < 0.05 |
| Times of alcohol consuming: 0 vs. ≥11 | > 0.999 |
| Times of alcohol consuming: 1-10 vs. ≥11 | 0.066 |
| How long has it been since you were first diagnosed with coronary heart disease: ≤6 months vs. >6 months, ≤1 year | < 0.05 |
| How long has it been since you were first diagnosed with coronary heart disease: ≤6 months vs. >1 year, ≤3 years | 0.009 |
| How long has it been since you were first diagnosed with coronary heart disease: ≤6 months vs. >5 years | < 0.05 |
| How long has it been since you were first diagnosed with coronary heart disease: >6 months, ≤1 year vs. >1 year, ≤3 years | < 0.05 |
| How long has it been since you were first diagnosed with coronary heart disease: >6 months, ≤1 year vs. >5 years | 0.014 |
| How long has it been since you were first diagnosed with coronary heart disease: >1 year, ≤3 years vs. >5 years | < 0.05 |

**Supplement** Table 2. Knowledge dimension

| **Knowledge** | **Correctness, n (%)** |
| --- | --- |
| 1. After PCI for myocardial infarction, care must be taken to prevent the recurrence of myocardial infarction. | 316 (66.39) |
| 2. After PCI, patients should always carry nitroglycerin with them. | 293 (61.55) |
| 3. Stent thrombosis is a serious complication after PCI, and it was mostly found in the early post-PCI period (0 to 30 days). | 61 (12.82) |
| 4. If you have already undergone PCI, you don’t need to take medication for a long-term period. | 418 (87.82) |
| 5. You don’t need to stop smoking if you have undergone PCI. | 441 (92.65) |
| 6. You don’t need regular follow-up consultations if you have no uncomfortable conditions after PCI. | 136 (28.57) |
| 7. For patients with a history of hypertension and with persistent systolic blood pressure >130 mmHg after PCI, it should be considered medication to lower blood pressure. | 208 (43.70) |
| 8. Post-PCI hypotension, as evidenced by a systolic blood pressure < 90 mmHg. | 389 (81.72) |
| 9. After PCI, moderate exercise is needed if the condition is stable. | 453 (95.17) |
| 10. After PCI, attention should be paid to an appropriate diet and control of the intake of salt, sugar, and cholesterol. | 430 (90.34) |
| 11. Patients still need to monitor their pulse rate, blood pressure, and chest pain episodes on their own when discharged from the hospital after PCI. | 453 (95.17) |
| 12. Patients need to observe themselves for symptoms such as vomiting blood, black stools, bleeding spots on the skin, and bleeding gums, where the use of anticoagulants after PCI. | 324 (68.1) |

**Supplement** Table 3. Attitude dimension

|  | **Strongly agree, n (%)** | **Agree, n (%)** | **Neutral, n (%)** | **Disagree, n (%)** | **Strongly disagree, n (%)** |
| --- | --- | --- | --- | --- | --- |
| 1. You believe PCI effectively saves patients’ lives with acute myocardial infarction. | 245 (51.47) | 177 (37.18) | 23 (4.83) | 28 (5.88) | 3 (0.63) |
| 2. You believe that you have received effective treatment. | 270 (56.72) | 74 (15.55) | 115 (24.16) | 12 (2.52) | 5 (1.05) |
| 3. You are confident you can do a good job on postoperative self-management. | 174 (36.55) | 117 (24.58) | 103 (21.64) | 57 (11.97) | 25 (5.25) |
| 4. You think that it is very difficult to monitor your pulse and blood pressure every day after the intervention. | 248 (52.10) | 167 (35.08) | 20 (4.20) | 39 (8.19) | 2 (0.42) |
| 5. You think it is important to have a rational diet and exercise properly after PCI. | 334 (70.17) | 123 (25.84) | 19 (3.99) | 0 | 0 |
| 6. You think keeping a positive attitude after PCI is important. | 154 (32.35) | 157 (32.98) | 86 (18.07) | 70 (14.71) | 9 (1.89) |
| 7. Your fear of illness has diminished after PCI. | 218 (45.80) | 126 (26.47) | 102 (21.43) | 11 (2.31) | 19 (3.99) |
| 8. You are concerned that you may experience a recurrence or postoperative complications in the future. | 23 (4.83) | 76 (15.97) | 179 (37.61) | 198 (41.60) | 0 |
| 9. You think that the PCI you have undergone has added a burden on your family. | 149 (31.30) | 105 (22.06) | 104 (21.85) | 100 (21.01) | 18 (3.78) |
| 10. You think that your illness and the surgery you received have negatively affected your interpersonal relationships. | 281 (59.03) | 124 (26.05) | 46 (9.66) | 25 (5.25) | 0 |

**Supplement** Table 4. Practice dimension

|  | Very conforming, n (%) | Conforming, n (%) | Moderate, n (%) | Non-conforming, n (%) | Very non-conforming, n (%) |
| --- | --- | --- | --- | --- | --- |
| P1. You will proactively monitor your health indicators: pulse rate, blood pressure, weight, etc. | 132 (27.73) | 98 (20.59) | 37 (7.77) | 177 (37.18) | 32 (6.72) |
| P2. After PCI, you will take regular medication for a long period as prescribed by your doctor | 230 (48.32) | 140 (29.41) | 49 (10.29) | 44 (9.24) | 13 (2.73) |
| P3. After PCI, you will follow your doctor’s instructions and attend regular follow-up consultations | 236 (49.58) | 121 (25.42) | 20 (4.20) | 68 (14.29) | 31 (6.51) |
| P5. You will seek medical attention if you have any symptoms of discomfort. | 282 (59.24) | 182 (38.24) | 9 (1.89) | 3 (0.63) | 0 |
| P6. After PCI, you will follow your doctor’s instructions and maintain an appropriate level of exercise for a long period. | 275 (57.77) | 151 (31.72) | 31 (6.51) | 19 (3.99) | 0 |
| P7. After PCI, you will follow your doctor’s advice and stop smoking thoroughly. | 294 (61.76) | 126 (26.47) | 56 (11.76) | 0 | 0 |
| P8. After PCI, you will never drink alcohol again | 294 (61.76) | 155 (32.56) | 27 (5.67) | 0 | 0 |
| P9. After PCI, you will pay attention to having a rational diet and rational nutrition and control the intake of salt, sugar, and cholesterol. | 298 (62.61) | 144 (30.25) | 34 (7.14) | 0 | 0 |
| P10. After PCI, you will pay close attention to your mental health. | 170 (35.71) | 240 (50.42) | 58 (12.18) | 8 (1.68) | 0 |
| P11. You will be proactive in learning about PCI and postoperative self-management. | 284 (59.66) | 131 (27.52) | 59 (12.39) | 2 (0.42) | 0 |
