# Supplementary figures and images for: Knowledge, attitude, and practice toward postoperative self‐management among patients after percutaneous coronary intervention: A structural equation modeling analysis
Source: Clin Cardiol. 2024 Mar 15;47(3):e24232. doi: 10.1002/clc.24232 (PMC10943248; doi:10.1002/clc.24232)

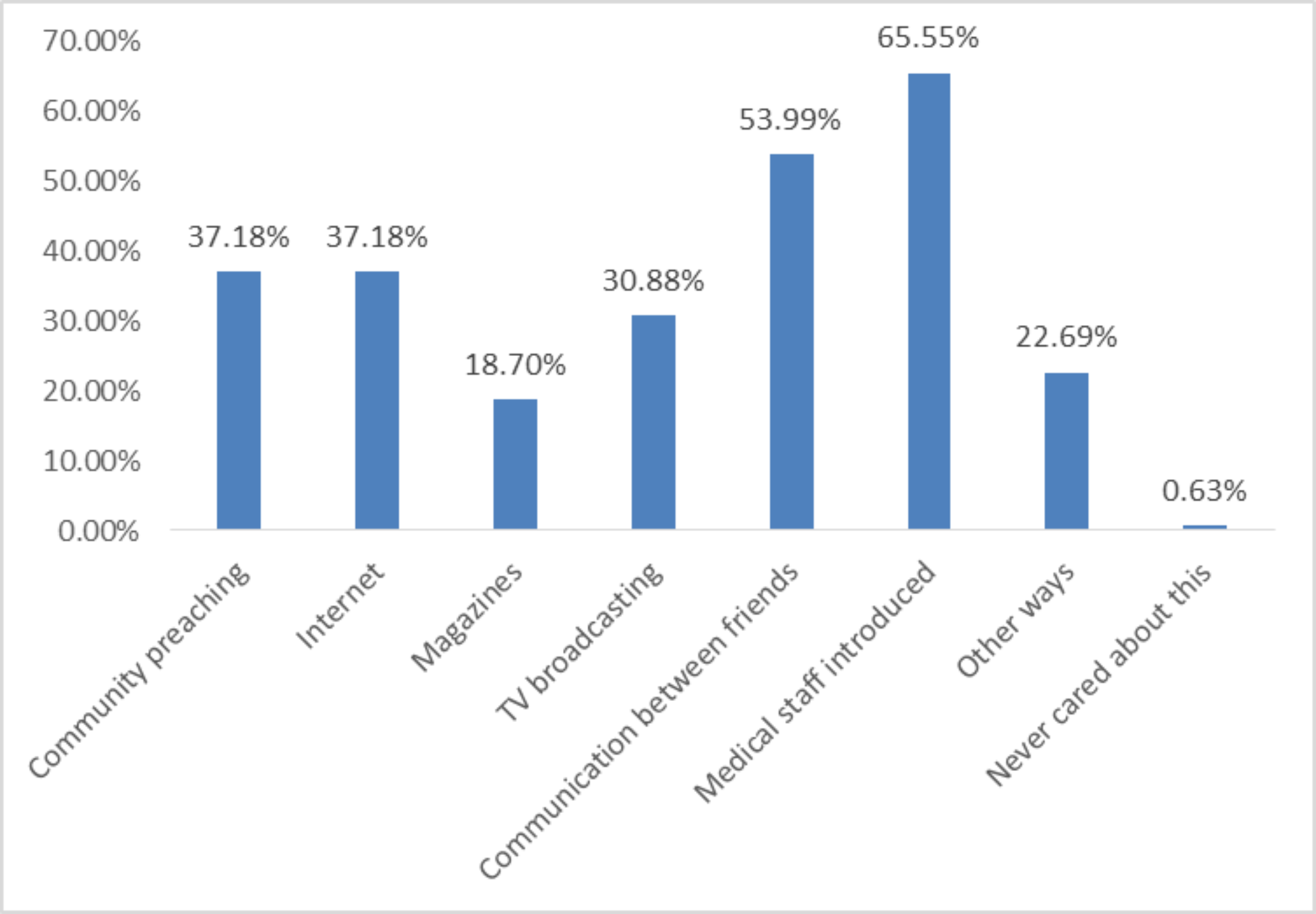

Supplement: Supplementary file 2 — Supplement Figure 1. Ways to acquire knowledge. [file CLC-47-e24232-s002.tiff]

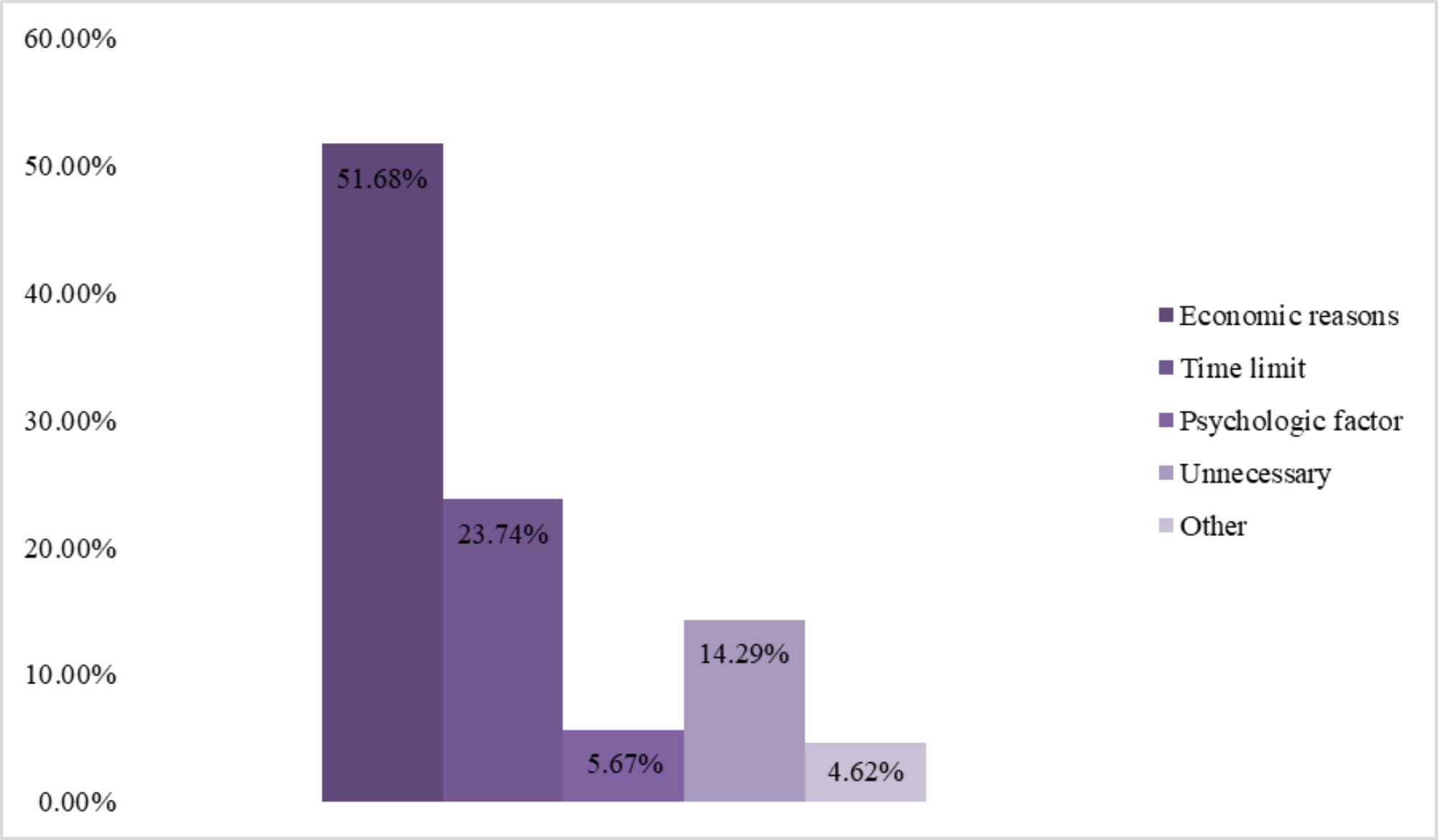

Supplement: Supplementary file 3 — Supplement Figure 2. Possible reasons for not being able to return to the hospital on time. [file CLC-47-e24232-s001.tiff]
